# HIV-related lung cancer in Uganda: a cohort study

**DOI:** 10.1186/s13027-022-00439-x

**Published:** 2022-06-06

**Authors:** Joseph Baruch Baluku, Naghib Bogere, Sharon Namiiro, Victoria Walusansa, Irene Andia-Biraro, William Worodria, Bruce Kirenga

**Affiliations:** 1 Makerere University Lung Institute, PO Box 26343, Kampala, Uganda; 2 Kiruddu National Referral Hospital, Kampala, Uganda; 3 Uganda Cancer Institute, Kampala, Uganda; 4 Makerere University College of Health Sciences, Kampala, Uganda; 5 Mulago National Referral Hospital, Kampala, Uganda

**Keywords:** Lung cancer, HIV, Uganda, Clinical features, Survival, Africa

## Abstract

**Background:**

There are few reports on lung cancer among people with HIV (PWH) in Sub-Saharan Africa. In this report, we describe a cohort of PWH and lung cancer at the Uganda Cancer Institute.

**Methods:**

This retrospective cohort of PWH and lung cancer was managed at the Uganda Cancer Institute between 2008 and 2018. Sociodemographic and clinical data were abstracted from the patient charts. The median survival from diagnosis to death, loss-to-follow up or 31st December 2018, was estimated.

**Results:**

There were 18 people with HIV and lung cancer. The median (interquartile range, IQR) age was 49.5 (38.8–56.0) years, 11 (61.1%) were women and 5 (27.8%) were smokers. Of the 18 PWH, 13 (72.2%) were on antiretroviral therapy and the median (IQR) CD4 count (n = 13) was 380 (243.5–595) cells per mm^3^. Difficulty in breathing (88.9%), chest pain (78.6%, n = 11), cough (76.5%, n = 17) and weight loss (72.2%) were the commonest symptoms while pleural effusions were observed in 12 (66.7%). In this cohort, 8 (44.4%) were presumptively treated for tuberculosis before the diagnosis of lung cancer. Seven (38.9%) had an Eastern Cooperative Oncology Group performance status of 3. Non-small cell lung cancer was the predominant histological type observed in 17 (94.4%) of whom 14 (82.4%) had adenocarcinoma. Majority of PWH had stage IV disease (88.9%). The median (IQR) survival was 3.3 (1.1–13.2) months and all were either dead (72.2%) or lost-to-follow up (27.8%) at five years from diagnosis.

**Conclusion:**

People with HIV and lung cancer in Uganda report low rates of smoking, present with advanced disease and post very poor survival rates. There is need for biomarkers for early detection of lung cancer in HIV.

## Background

Lung cancer is the leading cause of death from cancer accounting for 1.8 million global deaths in 2020 [1]. The incidence of lung cancer is on the rise in several regions of sub-Saharan Africa (SSA) owing to an increase in cigarette smoking rates and environmental pollution [2]. SSA is also home to two-thirds of people with HIV infection (PWH) who have a threefold higher incidence of lung cancer than people without HIV [3, 4]. Unfortunately, there are no clear screening recommendations for lung cancer among PWH [5]. A meta-analysis of cohort studies from the United States of America (USA) suggests that people with HIV-associated lung cancer have a 48% higher risk of mortality than HIV negative people with lung cancer [6]. However, there are few cohort studies of PWH and lung cancer from SSA. Some reports from this region report that PWH have a lower or no significant risk of lung cancer than HIV negative individuals [7–9]. Nonetheless, PWH and lung cancer seem to present with advanced disease and have poor performance status at diagnosis [10].This highlights the need to characterise the epidemiology, risk factors, clinical presentations and treatment outcomes of PWH and lung cancer in Africa.

In Uganda, 17% of people with lung cancer have HIV infection [11]. The incidence of lung cancer appears to increase later on (2–5 years) during HIV treatment for PWH [12]. In this study, we describe sociodemographic and clinical features and outcomes of PWH and lung cancer drawn from a 10-year cohort of people with lung cancer in Uganda [13].

## Methods

### Study design, population, and setting

This was a secondary analysis of data from a retrospective 10-year cohort of people with histologically confirmed lung cancer at the Uganda Cancer Institute (UCI) between 2008 and 2018 [13]. In this analysis, we included all people with HIV infection in the primary cohort. The Uganda Cancer Institute is a tertiary care facility which is the largest cancer treatment centre in Uganda. It is also designated as the centre of excellence for cancer care in the East African Region. Each year, about 4,000 people receive inpatient and outpatient cancer care at the UCI.

### Data collection

In the primary study, data were abstracted from patient charts using a data abstraction form. Specifically, baseline socio-demographic data on age, sex, and any history of smoking and/or alcohol use were collected. Clinical variables were baseline patient’s presenting complaints, comorbid conditions, clinical signs, and baseline laboratory findings (full haemogram, lactate dehydrogenase, liver transaminases, serum creatinine, urea, albumin, bilirubin, and tumour markers). Other variables collected were the histological diagnosis of lung cancer, cancer stage, sites of metastasis, modalities of treatment, and vital status (dead, alive or lost to follow up by 31st December 2018). A full description of the study procedures is available from the primary study [13].

### Data analysis

Data were entered in Microsoft Excel and analysed with STATA version 16.0. We used descriptive statistics to describe the characteristics of PWH and lung cancer. As such, proportions and medians with corresponding interquartile ranges (IQR) were used.

## Results

Of 207 participants in the primary study, 18 (8.7%) had HIV infection and were all included in the current analysis.

### Socio-demographic characteristics

The median (interquartile range, IQR) age was 49.5 (38.8–56.0) years, 11 (61.1%) were female and 5 (27.8%) were smokers. Table 1 shows the socio-demographic characteristics.

**Table 1:** Sociodemographic characteristics of PWH and lung cancer

Characteristic	Frequency (n = 18) (n%)
Age, median (interquartile range, IQR), years	49.5 (38.8–56.0)
Female	11 (61.1)
*Nationality*	
Ugandan	17 (94.4)
Rwandese	1 (5.56)
*Region*	
Central	9 (50.0)
Eastern	3 (16.7)
Northern	2 (11.1)
Western	3 (16.7)
Other	1 (5.6)
Smoking history	5 (27.8)
Smoking duration (years), Median (IQR)	20 (5)
Alcohol use	10 (55.6)
Family history of cancer	1 (5.6)

### Clinical characteristics of PWH and lung cancer in Uganda

Of the 18 PWH, 13 (72.2%) were on antiretroviral therapy (ART) (all on first-line regimens). The median (IQR) CD4 count (n = 13) was 380 (243.5–595) cells per mm^3^. Difficulty in breathing (88.9%), chest pain (78.6%, n = 11), cough (76.5%, n = 17) and weight loss (72.2%) were the commonest symptoms. Pleural effusions were observed in 12 (66.7%) and 1 (5.6%) had superior vena cava obstruction syndrome. In this cohort, 8 (44.4%) were presumptively treated for tuberculosis before the diagnosis of lung cancer. Seven (38.9%) had an Eastern Cooperative Oncology Group (ECOG) performance status of 3 and the median (IQR) body mass index was 20.8 (19.0–23.2) kilograms/metres^2^ (n = 7). Non-small cell lung cancer was the predominant histological type observed in 17 (94.4%), of whom 14 (82.4%) had adenocarcinoma and 3 (17.6%) had squamous cell carcinoma. The majority had stage IV disease (88.9%). Table 2 shows other clinical characteristics.

**Table 2:** Clinical characteristics of PWH and lung cancer in Uganda

Clinical characteristic	Frequency (n = 18), n(%)
*Symptoms*	
Haemoptysis (n = 17)	5 (29.4)
Cough (n = 17)	13 (76.5)
Weight loss	13 (72.2)
Difficulty in breathing	16 (88.9)
Chest pain (n = 14)	11 (78.6)
*Comorbidities*	
Hypertension	3(16.7)
Diabetes mellitus (n = 17)	1 (5.9)
Asthma (n = 17)	2 (11.8)
Chronic kidney disease (n = 17)	1 (5.9)
Herpes Zoster	1 (5.6)
Kaposi’s sarcoma	1 (5.6)
Previous confirmed pulmonary tuberculosis (TB) (n = 16)	2 (12.5)
Treatment for unconfirmed TB before diagnosis of lung cancer	8 (44.4)
Antiretroviral therapy*	13 (72.2)
*ECOG performance status*	
1	5 (27.8)
2	6 (33.3)
3	7 (38.9)
*Lung cancer stage*	
3B	1 (5.9)
4A	7 (38.9)
4B	9 (50.0)
Extensive disease (for small cell lung cancer)	1 (5.9)
*Site of metastasis*	
Liver (n = 17)	2 (11.8)
Contralateral lung (n = 17)	6 (35.3)
Brain (n = 16)	1 (6.3)
Bone (n = 16)	3 (18.8)
Pleura (n = 16)	12 (75.0)
*Laboratory parameters, median (interquartile range)*	
Albumin, grams per litre (g/l)	32.6 (5.6)
Total Protein (g/l)	70.8 (6.2)
Alkaline phosphatase, international units per litre (IU/L)	137 (107.6)
Alanine transferase (IU/L)	17.8 (13.7)
Aspartate aminotransferase (IU/L)	25.1 (21.1)
Gamma glutamyl transpeptidase (IU/L)	76.8 (77.0)
Serum creatinine, micromoles per litre (µmol/L)	66 (26.5)
Total bilirubin (µmol/L)	4.6 (7.0)
Direct Bilirubin (µmol/L)	2.67 (4.8)
Lactate dehydrogenase (units per litre)	248 (171.0)
Neutrophil-leucocyte ratio	3.5 (4.6)
Monocyte-leucocyte ratio	0.4 (0.3)
Thrombocyte-leucocyte ratio	147.7 (251.6)
Haemoglobin (grams per decilitre)	10.2 (3.7)
*Tumour markers*	
CEA (micrograms per litre), median (IQR), (n = 8)	11.3 (112.9)
CA 19-9 (units per millilitre), median (IQR), (n = 6)	2.8 (26.5)
CA 125 (units per millilitre), median (IQR), (n = 6)	123.7 (382.7)

*ECOG* Eastern Cooperative Oncology Group, *CEA* Carcinoembryonic antigen, CA 19-9-Carbohydrate antigen 19-9, CA 125-Cancer antigen 125; *of whom five were on tenofovir/lamivudine/efavirenz, three were on zidovudine/lamivudine/nevirapine, three were on zidovudine/lamivudine/efavirenz, one was on tenofovir/lamivudine/nevirapine and another one was on abacavir/lamivudine/efavirenz

### Modalities of treatment and lung cancer survival among PWH and lung cancer

**Fig. 1:**
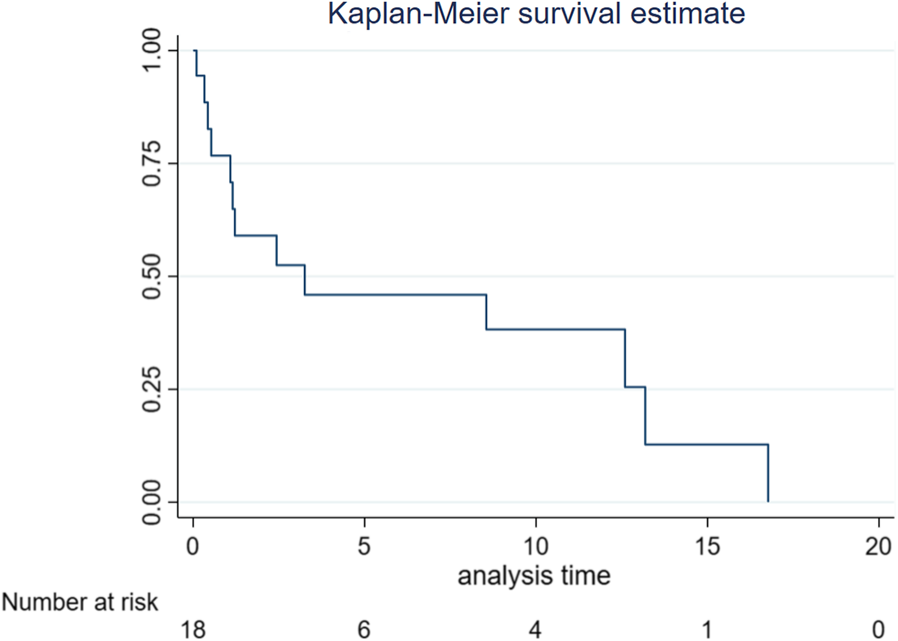
Kaplan–Meier survival curve for people with HIV and lung Cancer in Uganda

Chemotherapy was administered in 8 (44.4%), 6 (33.3%) received biological agents and 1 (5.6%) received radiotherapy. Some 7 (38.9%) died or were lost to follow up before starting any therapies. The median (IQR) survival was 3.3 (1.1–13.2) months and all were either dead (72.2%) or lost-to-follow up (27.8%) at five years from diagnosis. Figure 1 shows the Kaplan–Meier survival curve for this population.

## Discussion

In this study, we describe the first cohort of PWH and lung cancer in Uganda. We found that majority of these individuals were non-smokers, had advanced disease and very poor survival rates. Moreover most were presumptively treated for unconfirmed tuberculosis before the diagnosis of lung cancer. The predominant histological type was non-small cell lung cancer (adenocarcinoma).

The mechanisms by which HIV increases the risks of lung cancer are not fully understood. HIV infection may increase the expression of oncogenes and downregulation of tumour suppressor genes [14]. Other possible mechanisms include chronic inflammation due to HIV and other lung infections, immune suppression and traditional risk factors of lung cancer (smoking and chronic obstructive pulmonary disease) which are more prevalent among PWH [15].

Similar to our findings, PWH and lung cancer were relatively young, had an advanced stage of lung cancer, and poor ECOG status in South Africa [10]. Several cohorts in high income countries report similar socio-demographic and clinical characteristics with our study. PWH and lung cancer are mostly middle aged (38 to 49 years), present mostly with cough, weight loss, dyspnoea, and pleural effusions [16]. Further, about 67–86% have non-small cell lung cancer and predominantly present with stage IV disease (50–68%) [16]. There is need for sensitive biomarkers for early diagnosis of lung cancer among PWH. In our cohort, a significant number of PWH were treated with anti-TB therapy before diagnosis of lung cancer despite the lack of mycobacterial evidence of TB. This contributes to delays of lung cancer diagnosis.

The low survival rates reported in this study are similar to other cohorts that report median survival of only 3–6 months [16]. This can be attributed to late stage at presentation and poor performance status. Late presentation of cancer is observed in non-small cell lung cancer [17], breast cancer [18], cervical cancer [19], prostate cancer [20], and head and neck cancers [21] in Uganda. Moreover, 35% and 41% of people with cancer in Uganda delay treatment initiation and miss clinic appointments, respectively, due to lack of money for drugs, transportation and accommodation [22]. There is an urgent need to build diagnostic capacity and address barriers to early diagnosis and treatment initiation among people with cancer in Uganda on the overall.

It was interesting to note that majority of the PWH were on ART and were apparently immune competent (CD4 > 200 cells per mm^3^). The effect of ART and the immune status on lung cancer outcomes needs to be evaluated by larger cohorts. A key limitation of this study is the lack of data on viral loads. This would have otherwise characterised the viral load status of PWH and lung cancer in Uganda. However, these data were not available since participants received ART from other centres. Also, the sample size was too small to perform any inferential statistics for predictors of survival. Moreover, this was a single centre cohort, and this limits generalisability. Nonetheless, UCI registers most people with lung cancer in Uganda and our cohort is likely to be representative of PWH and lung cancer in Uganda.

## Conclusion

In this cohort of PWH in Uganda, most PWH with lung cancer have similar clinical presentation to PWH from high income countries. However, they presented with advanced lung cancer, poor performance status and posted very poor survival rates. Most were presumptively treated for unconfirmed TB before the lung cancer diagnosis. There is need to build nationwide capacity for early detection of lung cancer with a particular focus on differentiating lung cancer and pulmonary TB among PWH.

## Data Availability

The datasets used and/or analyzed during the current study are available from the corresponding author on reasonable request.

## Abbreviations

PWH: People with HIV
TB: Tuberculosis
UCI: Uganda Cancer Institute
ART: Antiretroviral therapy
ECOG: Eastern cooperative oncology group
CEA: Carcinoembryonic antigen
CA 19: 9-Carbohydrate antigen 19-9
CA 125: Cancer antigen 125
